# Repeated Extraneous Introductions of Cholera, Thailand, 2007–2025

**DOI:** 10.3201/eid3206.251747

**Published:** 2026-06

**Authors:** Kazuhisa Okada, Amonrattana Roobthaisong, Warawan Wongboot, Pawinee Doung-ngern, Wichan Bhunyakitikorn, Pilailuk A. Okada, Thanee Wongchai, Witaya Swaddiwudhipong, Tetsuya Iida, Shigeyuki Hamada

**Affiliations:** The University of Osaka Thailand–Japan Research Collaboration Center on Emerging and Re-emerging Infections, Mueang, Thailand (K. Okada, A. Roobthaisong); The University of Osaka Research Institute for Microbial Diseases, Suita, Japan (K. Okada, T. Iida, S. Hamada); Ministry of Public Health, Mueang (W. Wongboot, P. Doung-ngern, W. Bhunyakitikorn, P.A. Okada); Maesot General Hospital, Maesot, Thailand (T. Wongchai, W. Swaddiwudhipong)

**Keywords:** *Vibrio cholerae*, cholera, bacteria, Thailand, genomic analysis, cross-border transmission, phylogenetic tree

## Abstract

Genomic analyses identified 4 seventh pandemic *Vibrio cholerae* El Tor clades in Thailand (2007–2025). Closely related to other South Asian strains, the clades reveal that repeated cross-border introductions, rather than local persistence, drive outbreaks. Our findings highlight the importance of genomic surveillance for monitoring transmission and informing regional control strategies.

Cholera, caused by *Vibrio cholerae* O1, is a potentially life-threatening diarrheal disease and remains a major global public health threat (*1*). In recent years, large-scale outbreaks have occurred in several countries in Africa and the Middle East, including Sudan and Yemen, where conflict, population displacement, and limited access to clean water have contributed to increased transmission (*2*,*3*). South Asia, particularly the Bengal Delta, serves as a hub for genetically diverse pandemic strains and contributes to their global dissemination (*4*,*5*). Outbreak occurrence and severity are influenced by environmental and socio-economic factors, and *V. cholerae* can persist in aquatic environments, although long-term local persistence varies by region (*6*–*9*).

Cholera has resurged periodically in Thailand, notably from 2007, when a large-scale outbreak affected 50 provinces, peaking in northeastern regions between September and November. Analyses during 2007–2010 using pulsed field gel electrophoresis and multilocus variable-number tandem repeat analysis indicated that those outbreaks were driven by repeated introductions of *V. cholerae* O1, particularly in border areas (*10*). Independent evidence further suggested regional transmission or shared infection sources among Thailand, Laos, and Vietnam. Previous studies have revealed high clonal diversity among Thailand isolates collected during 1999–2002 (*11*), whereas isolates of the El Tor serotype Ogawa strain from 2007 displayed a distinct ribotype, consistent with the introduction of a new clone in northeastern Thailand. Despite those insights, understanding of the genomic relationships between historical and more recent Thailand isolates and global pandemic strains remains incomplete.

## The Study

**Figure 1:**
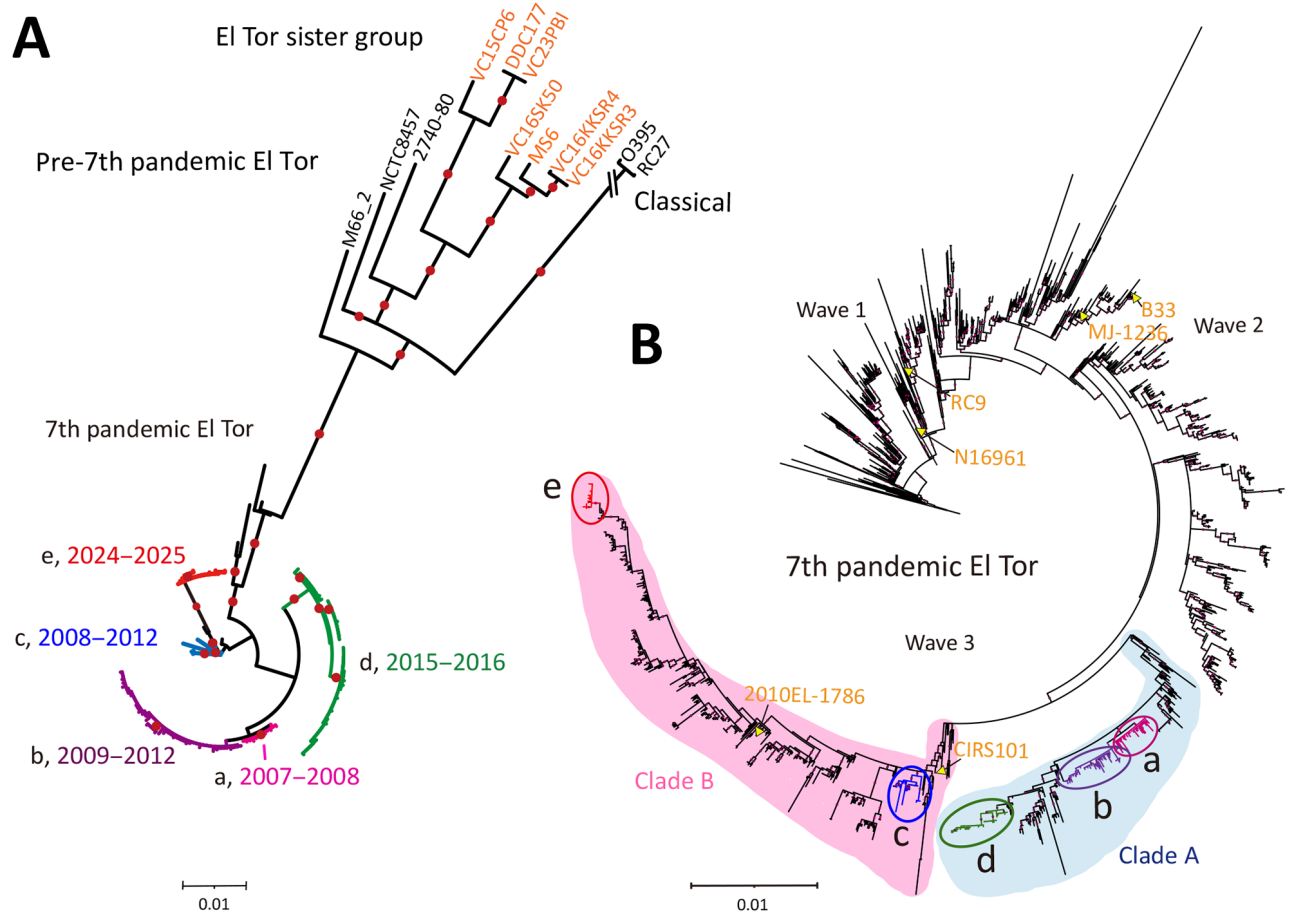
Maximum-likelihood phylogenetic trees of *Vibrio cholerae* O1 isolates reconstructed for study of repeated extraneous introductions of cholera, Thailand, 2007–2025. A) Phylogenetic relationships of 157 Thailand isolates and 11 representative O1 reference strains. Seven Thailand isolates (orange) belong to the El Tor Sister group lineage. The 7th pandemic El Tor lineage is divided into 5 distinct clusters (a–e), color-coded by isolation period: red-purple (cluster a, 2007–2008), blue-purple (cluster b, 2009–2010), blue (cluster c, 2011–2012), green (cluster d, 2015–2016), and red (cluster e, 2024–2025). B) Detailed phylogeny of the 7th pandemic El Tor lineage incorporating 150 Thailand isolates and 1,425 global genomes. Yellow labels explicitly identify the global reference strains (e.g., N16961, CIRS101) used in the analysis to provide genomic context for the Thailand clades. Light blue shaded region highlights clade A; light pink shaded region highlights clade B. Both were selected for molecular clock analysis. Outbreak-associated Thailand clades (TH1–TH4) are nested within these broader genomic groups. Both trees were reconstructed using maximum-likelihood estimation based on high-quality core genome single-nucleotide polymorphisms and visualized using Interactive Tree of Life (https://itol.embl.de). Dots on the branches indicate bootstrap values >80%.

We analyzed 498 *Vibrio cholerae* O1 isolates from Thailand collected from 2007 through April 2025 and selected 157 representative isolates for whole-genome sequencing: 85 from 2007–2012, 52 from 2015–2016, and 20 from 2023–2025 (including 72 newly sequenced in this study). We chose our isolate pool to reflect the genetic diversity observed in the multilocus variable-number tandem-repeat analysis and to ensure variation in the collection site and isolation period (Appendix 1 Tables 1, 2). Cholera case numbers and their geographic distribution in Thailand, obtained from reports by the Division of Epidemiology (Appendix 2 Figure 1), showed a marked nationwide decline in outbreak size and frequency. From 2017 to early 2025, the annual number of cases in Thailand remained <20 (mean 7.2), and no large-scale, nationwide outbreaks occurred during that period. We included in our analyses isolates from smaller clusters near the northwestern border and sporadic cases from samples collected in late 2024 and early 2025.

We conducted a series of analyses to investigate the evolutionary dynamics of *V. cholerae* O1 isolates in Thailand during 2007–2025, clarifying their origins, transmission patterns, and the relationships between isolates from Thailand and those from outside the country. We sequenced genomic DNA from the isolates using an Illumina MiSeq platform (https://www.illumina.com) and mapped to the *V. cholerae* N16961 reference genome. For global comparison, we curated *V. cholerae* O1 genomes from the National Center for Biotechnology Information Sequence Read Archive (https://www.ncbi.nlm.nih.gov/sra) and GenBank on the basis of published studies (Appendix 1 Table 3), ensuring balanced geographic and temporal representation. We quality-filtered data from the Sequence Read Archive using fastp software (https://github.com/opengene/fastp; -q parameter), retaining only high-quality sequences. We then identified core genome single-nucleotide polymorphisms (SNPs) using Snippy (https://github.com/tseemann/snippy), excluding regions associated with prophages (identified using PHAST [https://github.com/CshlSiepelLab/phast]), repeats (identified using NUCmer [https://github.com/nf-core/modules/tree/master/modules/nf-core/nucmer]), and recombination (identified using Gubbins [https://github.com/nickjcroucher/gubbins]). We masked these regions using VCFtools (https://github.com/vcftools/vcftools) before phylogenetic analysis. We reconstructed maximum-likelihood phylogenetic trees using RAxML software (https://cme.h-its.org/exelixis/web/software/raxml) and analyzed temporal signals using root-to-tip regression. We estimated divergence times using Bayesian molecular clock analysis. Pairwise SNP comparisons identified the 5 closest non-Thailand isolates for each outbreak cluster (Table). We used CholeraeFinder (Center for Genomic Epidemiology, https://www.genomicepidemiology.org) to detect virulence genes and mobile genetic elements. We also used Mantel tests as a complementary metric to assess broad congruence but drew the primary conclusions from the phylogenetic topologies to account for evolutionary structure.

**Table:** Single-nucleotide polymorphism differences between Thailand outbreak clusters and the most closely related non-Thailand strains from study of repeated extraneous introductions of cholera, Thailand, 2007–2025*

Lineage (cluster)	SNP range within cluster	Closest non-Thailand strains	Origin, country/year	SNPs from Thailand isolate
TH1 (a)	0–7	MAB004	Bangladesh/2005	7
		MAB006	Bangladesh/2005	8
		4488	India/2006	13
		THSTI_J4770	India/2004	13
		BGD005	Bangladesh/2007	13
TH1 (b)	0–7	MAB004	Bangladesh/2005	9
		MAB006	Bangladesh/2005	12
		4488	India/2006	17
		THSTI_J4770	India/2004	17
		BGD005	Bangladesh/2007	17
TH2 (c)	0–21	THSTI_K9398	India/2005	12
		MAB001	Bangladesh/2004	13
		CNRVC060089	India/2006	13
		CNRVC060333	India/Nepal/2006	13
		MBN17	India/2004	14
TH3 (d)	0–14	PCS-0023	Bangladesh/2010	6
		IDH-07956	India/2015	7
		BGD133	Bangladesh/2015	8
		HCIS-055B	Bangladesh/2013	10
		BGD128	Bangladesh/2015	10
TH4 (e)	0–5	DMAVC-8	Bangladesh/2022	5
		DMAVC-18	Bangladesh/2022	5
		DMAVC-17	Bangladesh/2022	7
		DMAVC-19	Bangladesh/2022	7
		DMAVC-4	Bangladesh/2022	8

*To identify the closest non-Thailand relatives, we calculated pairwise SNP distances for all 150 Thailand seventh pandemic isolates against the global dataset using snp-dists (https://github.com/tseemann/snp-dists). To capture the full genetic diversity, we compared each Thailand genome independently rather than using a single representative per cluster. For each cluster, we selected the 5 non-Thailand strains with the smallest SNP distances. SNP ranges within clusters reflect internal diversity, whereas differences from non-Thailand strains indicate potential geographic origins. SNP, single-nucleotide polymorphism.

**Figure 2:**
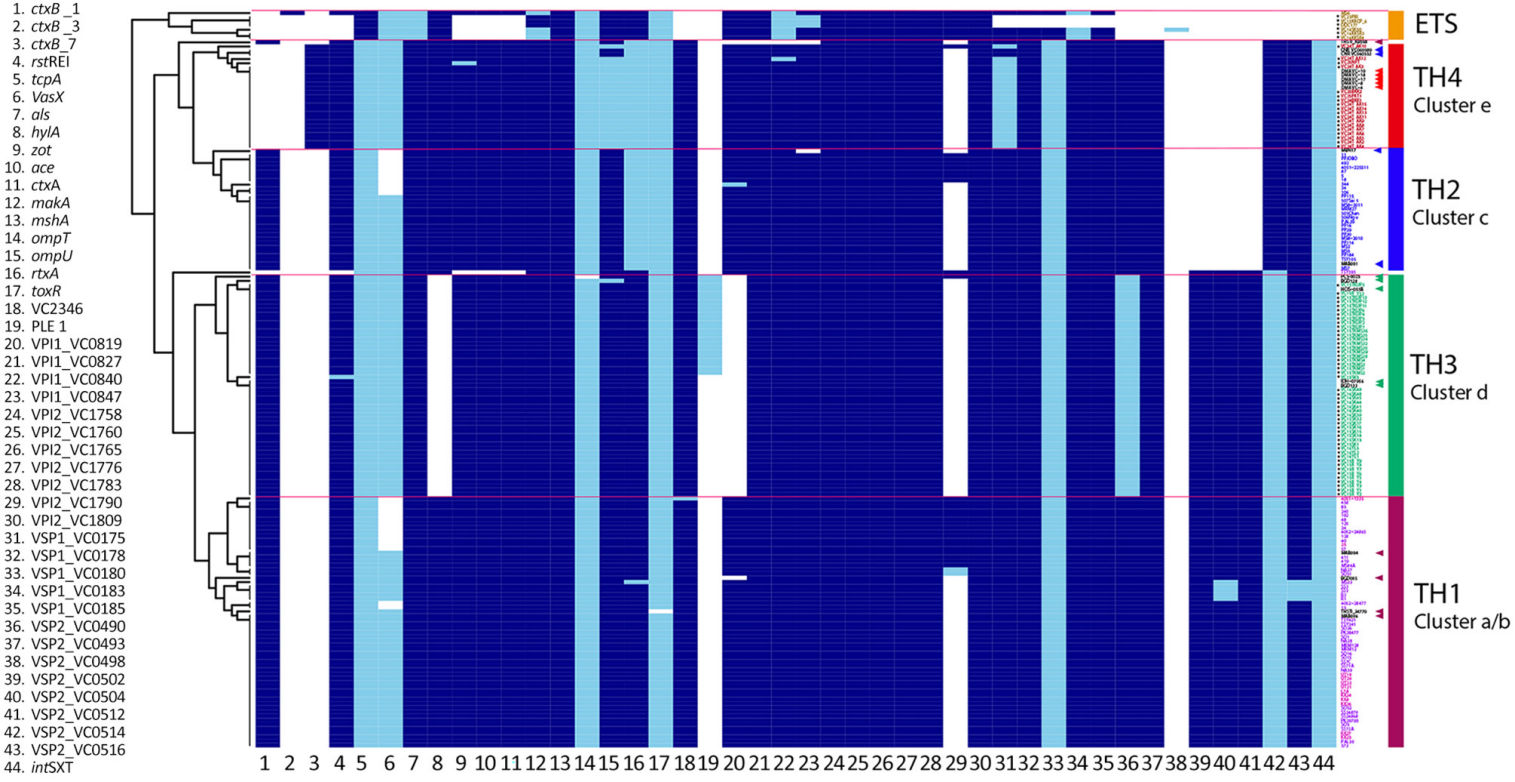
Phylogenetic relationships, genomic features, and outbreak cluster associations of *Vibrio cholerae* isolates from study of repeated extraneous introductions of cholera, Thailand, 2007–2025. Black dots indicate newly sequenced Thailand isolates in this study (n = 72) to distinguish them from reference strains. Virulence genes, integrative conjugative elements, and mobile genetic elements identified using CholeraeFinder (Center for Genomic Epidemiology, https://www.genomicepidemiology.org) in BLASTN mode (minimum coverage and identity 90%) (Appendix 2, Table 1). The blue cells in the heatmap represent the presence of genes, light blue cells indicate mutated genes, and blank cells signify the absence of genes. Phylogenetic relationships analyzed using a RAxML tree (https://cme.h-its.org/exelixis/web/software/raxml); genomic features are numerically coded (gene present = 1, gene absent = 0, mutated gene = 0.5). Thailand clades (TH1, TH2, TH3, and TH4) defined based on phylogenetic clustering and characteristic genomic features: TH1 (clusters a and b), TH2 (cluster c), TH3 (cluster d), and TH4 (cluster e). Arrowheads highlight the 5 closest non-Thailand isolates associated with each outbreak cluster, identified by pairwise single-nucleotide polymorphism comparisons. ETS, El Tor sister *V. cholerae* isolates.

Comparative genomic analysis of 157 Thailand isolates and global genomes identified 2 lineages: the seventh pandemic El Tor (7PET; 150 isolates) and the El Tor sister group (ST75; 7 isolates) (Figure 1). The 7PET clade comprised 4 distinct groups (TH1–TH4), corresponding to 3 outbreak periods (Figure 2). Period I (2007–2012) included TH1 (clusters a and b, Ogawa, CTX-3) and TH2 (cluster c, Inaba, CTX-3), with characteristic deletions in *Vibrio* seventh pandemic island [VSP] II. Period II (2015–2016) included TH3 (cluster d, Ogawa), characterized by CTX-3, VPI-1ΔVC0819, Δ*hlyA*, and variable presence of PLE1, which varied by the geographic region of cholera cases. Period III (2024–2025) was dominated by TH4 (cluster e, Ogawa), carrying CTX-3b (*ctxB7*), OmpU G325D, and deletions in VSP-II. Although deletions in canonical pathogenicity islands (e.g., VSP-II, VPI) could theoretically influence fitness, we observed no obvious association with clinical severity in the study dataset.

**Figure 3:**
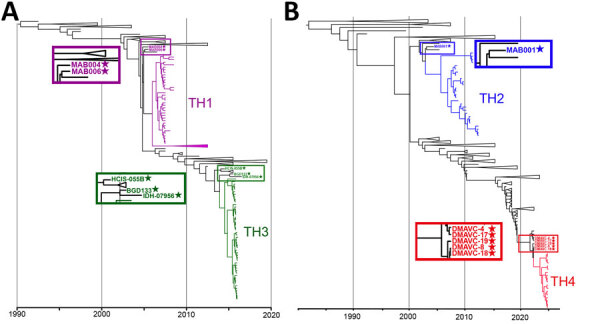
Temporal signal and divergence dating of Thailand *Vibrio cholerae* O1 isolates during the seventh pandemic from study of repeated extraneous introductions of cholera, Thailand, 2007–2025. Maximum clade credibility trees for clade A (panel A) and clade B (panel B) were reconstructed using a relaxed molecular clock (log-normal distribution) and a coalescent exponential population model. For clade A (n = 254; DRR189295 excluded), sampling years covered 1992–2019; for clade B (n = 417; ERR2265665 and DRR189283 excluded), sampling years covered 1992–2025. Both clades demonstrated strong temporal signals, confirmed by root-to-tip regression analysis (R^2^ > 0.91) (Appendix 2, Figure 2) and showed strong statistical support with effective sample size values >200. Temporal estimates suggest interregional transmission during the pandemic and highlight the preservation of specific genetic traits within Thailand populations, supporting the inferred lineage connections. Different colors indicate distinct clades, and stars indicate strains with characteristic genomic features.

We conducted temporal analysis (Appendix 2 Figure 2) and Bayesian molecular clock modeling (8,137 recombination-filtered SNPs) to estimate the temporal dynamics and evolutionary divergence of *V. cholerae* clades circulating in Thailand and worldwide (Figure 3). The clades exhibited clear evolutionary patterns over time, and the statistical analysis was well supported (effective sample size >200), indicating that the estimated divergence times were reliable. Thailand clades were closely related to other South Asia clades: TH1 corresponded to BD-2 (MAB004 and MAB006), TH2 to BD-1 (MAB001), TH3 to BD-2 (HCIS-055B, BGD133, and IDH-7956), and TH4 to BD-1.2 (DMAVC-4, -8, -17, -18, and -19). In Bangladesh, BD-1 and BD-2 have been the 2 most prominent clades during the past 2 decades, with BD-1.2 emerging more recently and responsible for a massive 2022 cholera outbreak (*12*).

## Conclusions

This study confirmed that Thailand experienced multiple independent waves of cholera during 2007–2025, primarily associated with repeated introductions of *V. cholerae* O1 strains from South Asia. Outbreak clades in Thailand during period I (2007–2012) were most closely related to the prominent BD-1 and BD-2 clades in Bangladesh. During period II (2015–2016), 3 distinct waves emerged in both the northwestern and southern regions of Thailand, and the southern isolates exhibited multilocus variable-number tandem-repeat analysis profiles identical to strains predominant in Mandalay, Myanmar, 6 months earlier (*13*), suggesting direct cross-border introductions. By period III (2024–2025), most cholera cases in Thailand were imported, primarily from Myanmar, which reported >2,000 cases in 2024 (*1*). Of note, CTX-3b isolates in Thailand were first detected in period III and belonged to the recently emerged BD-1.2 clade, responsible for a massive 2022 cholera outbreak in Bangladesh. Although our phylogenetic data highlight repeated introductions as the primary driver of outbreaks, the potential role of long-term environmental persistence cannot be ruled out owing to a lack of systematic environmental genomic data.

Our analysis also revealed non-7PET (El Tor sister, sequence type [ST] 75) isolates: 6 of the 7 we detected lacked cholera toxin genes and 1 was the toxigenic strain MS6 previously described in a border area in 2008 (*14*). During 2010–2020, ST75, rather than ST69, the major seventh pandemic lineage, emerged as the dominant clonal group in China and South Africa (*15*).

Although the geographic spread and origins of ST75 in Thailand remain unclear, our findings demonstrate the importance of continuous genomic surveillance for elucidating cholera transmission dynamics and informing outbreak response strategies. To translate our data into public health action, implementing routine genomic sequencing at border checkpoints and establishing a real-time data sharing framework with regional partners, particularly with Myanmar, Bangladesh, and India, is essential for the early detection and mitigation of future cholera waves in the South Asia region.

Appendix 1 Additional tables for repeated extraneous introductions of cholera, Thailand, 2007–2025.

Appendix 2 Additional figures for repeated extraneous introductions of cholera, Thailand, 2007–2025.
