# Supplementary material for: Repeated Extraneous Introductions of Cholera, Thailand, 2007–2025
Source: Emerg Infect Dis. 2026 Jun;32(6):991–6. doi: 10.3201/eid3206.251747 (PMC13245207; doi:10.3201/eid3206.251747)
Supplement: Appendix 2 — Additional figures for repeated extraneous introductions of cholera, Thailand, 2007–2025. [file 25-1747-Techapp-s2.pdf]

Article DOI: http://doi.org/10.3201/eid3206.251747

EID cannot ensure accessibility for supplementary materials supplied by authors. Readers who have difficulty accessing supplementary content should contact the authors for assistance.

Repeated Extraneous Introductions of Cholera, Thailand, 2007–2025

Appendix 2

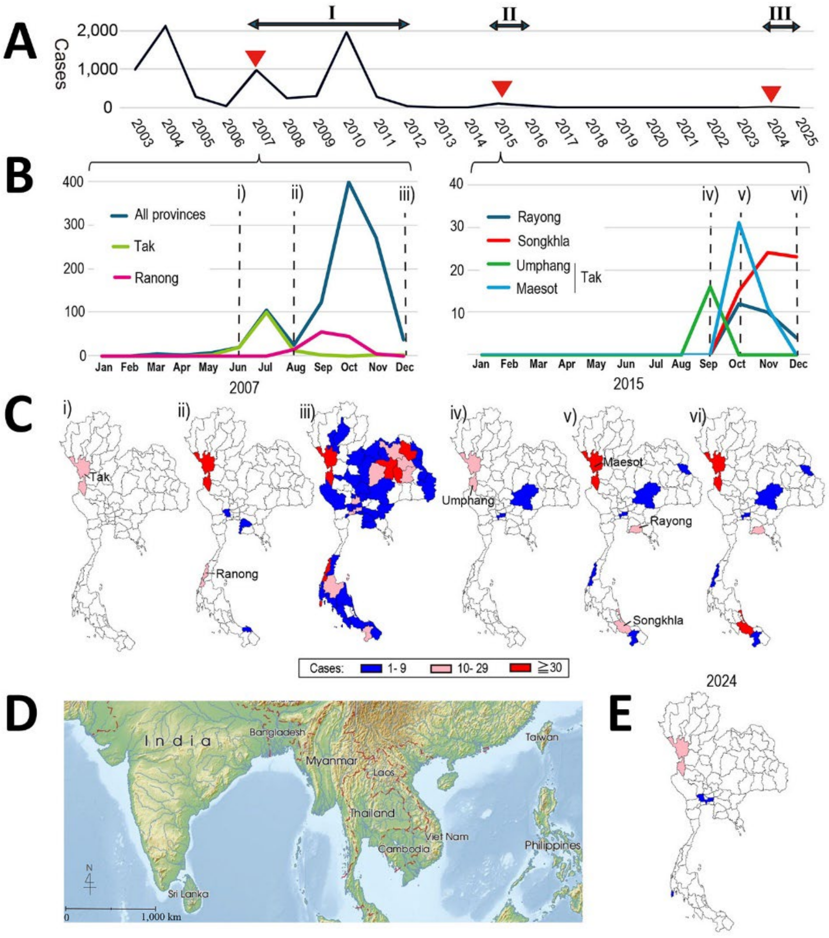

Appendix 2 Figure 1. Annual trends and monthly distribution of cases during epidemic periods. This figure illustrates the annual trends in case counts from 2003 to April 2025. (A) Sharp increases in cases,

identified as outbreak onsets, were observed in 2007 and 2015, marked by red arrowheads. In 2024, clusters of infections were reported in the northwestern border region, similar to those seen in 2007 and 2015. (B, C) Detailed monthly case counts and their distributions are presented for 2007 and 2015. The epidemic periods are defined as follows: Period I (2007–2012), Period II (2015–2016), and Period III (2024–2025). (D, E) Base map adapted from Mapswire (https://mapswire.com) under a Creative Commons (CC-BY 4.0) license.

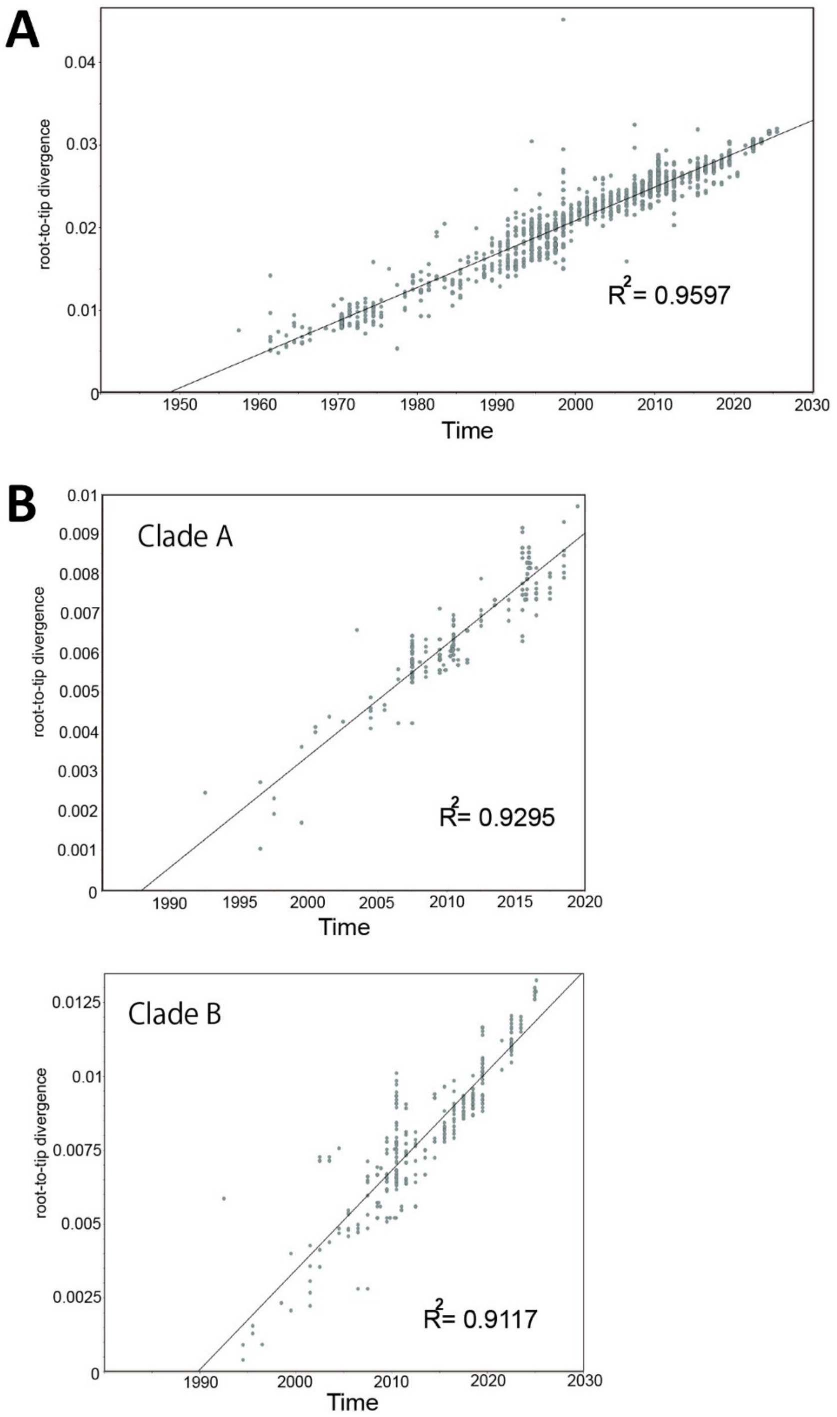

Appendix 2 Figure 2. Temporal signal analysis of the seventh pandemic *Vibrio cholerae* O1 lineage genomes using TempEST. (A) Root-to-tip regression for 1,575 genomes from the seventh pandemic lineage, showing a strong temporal signal (best-fitting root enabled) and an estimated Time to the Most

Recent Common Ancestor (TMRCA) of 1948.6 over a 68-year period. (B) Root-to-tip regression for clades A and B (254 and 417 genomes, respectively), as defined in Figure 1, showing a clear temporal structure within each clade.
